# Annotated mitochondrial genome with Nanopore R9 signal for *Nippostrongylus brasiliensis*

**DOI:** 10.12688/f1000research.10545.1

**Published:** 2017-01-19

**Authors:** Jodie Chandler, Mali Camberis, Tiffany Bouchery, Mark Blaxter, Graham Le Gros, David A Eccles

**Affiliations:** 1 Malaghan Institute of Medical Research, Wellington, New Zealand; 2 École Polytechnique Fédérale de Lausanne, Lausanne, Switzerland; 3 Institute of Evolutionary Biology, School of Biological Sciences, University of Edinburgh, Edinburgh, UK

**Keywords:** nanopore, MinION, parasite, mitochondria, de novo, phylogenetic, bioinformatics

## Abstract

*Nippostrongylus brasiliensis*, a nematode parasite of rodents, has a parasitic life cycle that is an extremely useful model for the study of human hookworm infection, particularly in regards to the induced immune response. The current reference genome for this parasite is highly fragmented with minimal annotation, but new advances in long-read sequencing suggest that a more complete and annotated assembly should be an achievable goal. We *de-novo* assembled a single contig mitochondrial genome from *N. brasiliensis* using MinION R9 nanopore data. The assembly was error-corrected using existing Illumina HiSeq reads, and annotated in full (i.e. gene boundary definitions without substantial gaps) by comparing with annotated genomes from similar parasite relatives. The mitochondrial genome has also been annotated with a preliminary electrical consensus sequence, using raw signal data generated from a Nanopore R9 flow cell.

## Introduction

*Nippostrongylus brasiliensis* is a parasitic nematode that naturally infects rodents. Its life cycle and morphology is comparable to *Necator americanus* and *Ancylostoma duodenale*, and it is thus an excellent murine model of human hookworm infection, a disease that affects approximately 700 million people worldwide ^1^. Like its human counterparts, *N. brasiliensis* L3 larvae infect the host through the skin and migrate to the lungs where they feed on red blood cells (unpublished study; Haem metabolism is a check-point in blood-feeding nematode development and resulting host anaemia; Bouchery T, Filbey K, Shepherd A, Chandler J, Patel D, Schmidt A, Camberis A, Peignier A, Smith AAT, Johnston K, Painter G, Pearson M, Giacomin P, Loukas A, Bottazzi M-E, Hotez P, Le Gros G), causing extensive haemorrhage and anaemia – both hallmarks of hookworm infections. The larvae are coughed up and swallowed to enter the gastrointestinal tract. The nematode matures into a sexually active adult in the small intestine where it secretes eggs that enter the environment via the hosts’ faeces. Larvae hatch, undergo two molts to become infective L3 larvae, which propagates the lifecycle ^2^. The immunology of *N. brasiliensis* infection has been studied extensively, and the parasite has been utilised as an inducer of potent Th2 responses in the lung and intestine, yielding important discoveries into cellular and molecular immune responses ^3– 6^. The *N. brasiliensis* model allows delineation of hookworm-induced immune profiles that could be targeted in drug or vaccine design, and provides a simple and well-characterised murine model in which to test these interventions for efficacy. To underpin these studies, a highquality reference genome is needed.

### Current reference genome

The most recent NCBI reference genome sequence for *N. brasiliensis* is a draft generated from Illumina HiSeq reads as part of the Wellcome Trust Sanger Institute (WTSI) 50 Helminth Genomes initiative ^7– 9^. It is 294.4 Mbp in total length, and highly fragmented (29,375 scaffolds with an N50 length of 33.5kb, and a longest scaffold of under 400kb). The *N. brasiliensis* reference genome would benefit from improvement, a goal that may be readily achieved with the advent of affordable long-read sequencing technologies.

### MinION sequencing

The Oxford Nanopore Technologies’ (ONT) MinION platform is improving at a rapid pace, with improvements in flow cell chemistry and base calling software announced frequently. In 2015, the median accuracy of double-stranded MinION reads, using R7.3 sequencing pores, was about 89% pores, sequenced at 60 bases per second with a yield of about 200 Mb ^10^. The quality and length of sequences generated from R7.3 pores was sufficient to create a single-contig assembly of the *Escherichia coli* K-12 MG1655 chromosome using nanopore reads alone, with consensus accuracy of 99.5% ^11^. An equimolar sample of *Mus musculus*, *E. coli* and *Enterobacteriophage lambda* DNA was sequenced in September 2016 on the International Space Station using R7.3 flow cells, producing approximately equal read counts for the different samples with a median accuracy of 83–92% for 2D reads across four runs ^12, 13^.

The recent introduction of R9 sequencing pores in June 2016, together with improved software for base-calling the generated signal trace at 250 bases per second ^14^, has improved the median accuracy of high-quality double-stranded reads to 95%, and yield to 800 Mb (personal communication, September 2016; MinION Analysis and Reference Consortium). Consensus accuracy for an *E. coli* K12 assembly consequently also increased to 99.96% ^15^. A rapid single-stranded sequencing kit was introduced in August 2016, reducing post-extraction sample preparation time to less than 15 minutes (see 16).

The R9.4 flow cell was commercially released by ONT a few months later in October 2016. This release brought together software and chemistry improvements that increased run flow cell yield into the gigabase range, and increased sequencing speed to 450 bases per second. Additional use cases for the MinION are evident with this increased yield: the R9.4 flow cells have already been used for sequencing human genomes using multiple flow cells, with observed yields of about 1–4Gb for each individual sequencing run ^17, 18^.

### Scientific justification

The mitochondrial genome is useful for epidemiology and population genetic analysis in nematodes, as it is rapidly evolving ^19, 20^. An average cell has 100–1000 mtDNA molecules, compared to two nuclear DNA molecules ^21^, and this stoichiometric excess facilitates analyses, especially where starting materials are limited. The strict maternal inheritance of the mitochondrial chromosome, coupled with a general lack of recombination in this haploid replicon permits inference of maternal lineages ^21– 23^. The ONT MinION can be deployed in infectious disease outbreak scenarios, and a "read until" methodology promises to make rapid, specific identification of known infectious agents possible. The technology has obvious utility in other areas of epidemiology and infection surveillance, and to enhance these applications it will be useful to develop the "read until" methodology to be able to detect a wider range of infectious agents from metagenomic sequencing. To do this, electronic signatures representing the MinION nanopore event signals could be used as a reference library to pre-screen raw signals from the pores before base calling. Here we present a complete mitochondrial genome for *N. brasiliensis*, assess its quality by gene prediction and phylogenetic analyses, and provide a validated electronic signal trace for the sequence.

This annotation represents the first hurdle in generating a complete genomic sequence for this model organism and provides crucial information for evolutionary and immunological studies. The rapid advancement of molecular technologies, such as qPCR, RNAseq, nanostring and high through-put sequencing, has given researchers the capacity to acquire an expansive array of new knowledge and insight into how genetic pathways function and interact at a molecular level. However, the lack of a complete annotated reference genome for *N. brasiliensis* thus far has restricted the full exploration into this important helminth.

## Methods and results

Genomic DNA was extracted from adult *N. brasiliensis* and sequenced on a MinION R9 flow cell. Reads from this sequencing run were then assembled, and the highest-coverage contig (mitochondrial DNA) was error-corrected and circularised for further analysis.

### DNA extraction and library preparation

*N. brasiliensis* was originally sourced from Lindsey Dent of the University of Adelaide, South Australia and has been maintained for 22 years by serial passage at the Malaghan Institute. Female Lewis rats were bred and used for the maintenance of the *N. brasiliensis* life cycle at 4 months of age (weight over 150g; housed in IVC caging and given *ad libitum* access to food and water). For the purposes of this study, one rat was infected with 4000 infective larvae. After 7 days, to allow the worms to mature to the adult stage in the small intestine, the rat was euthanized, and the small intestine dissected and flushed with PBS to harvest worms, as outlined in Camberis *et al.* ^2^. Ethics approval for the maintenance of the *N. brasiliensis* life cycle is overseen and approved by the Victoria University of Wellington Animal Ethics Committee.

The harvested *N. brasiliensis* were washed in PBS by centrifugation to remove cellular debris. The nematodes were frozen at -80°C bead-beaten, and DNA extracted using Qiagen DNeasy Blood and Tissue DNA extraction kit, yielding approximately 4 *µg* of high molecular weight double-stranded DNA (determined by the Quantus QuantiFluor dsDNA System). This DNA was treated with RNAse. Two sequencing libraries were made using the Oxford Nanopore 2D genomic DNA sequencing kit, yielding in total about 70ng of adapter-ligated sequencing library. No effort was made to specifically isolate mitochondrial DNA. The first preparation was loaded onto an R9 MinION flow cell and sequenced for 6 hours, and the second preparation was loaded onto the same flow cell and sequenced for an additional 36 hours. Pore occupancy at 30 minutes into the first run was about 25%, while pore occupancy at 30 minutes into the second run was about 80%.

### Whole-genome assembly with Canu

All FASTQ sequences (i.e. both 1D and 2D reads) were extracted from the base-called FAST5 files. These sequences were fed into Canu v1.3 ^24^ to generate assembled contigs. The contig with the highest coverage was a 19907 bp sequence with similarity to other nematode mitochondrial genomes (see Supplementary File 1). This sequence had 98% identity to an unannotated *N. brasiliensis* contig in the Wellcome Trust Sanger Institute (WTSI) *N. brasiliensis* assembly ^7^.

### Error correction and circularisation

Reads generated by WTSI (SRA ID: ERR063640) were mapped as pairs to the MinION mitochondrial contig using Bowtie2 ^25^ in local mode. At each location, one read was randomly sampled from those that mapped to that location, representing a reference-based digital normalisation to approximately 100X coverage (see Supplementary File 2). The differences between these normalised reads and the MinION contig were evaluated using a custom script, producing a corrected sequence based on the consensus read alignments. The mapping and correction process was repeated with BWA-MEM ^26^ on the corrected sequence (see Supplementary File 3) to identify additional variants that were missed by Bowtie2, due to multiple matches to duplicated regions.

Repeated sections of the linear contig (representing duplicated regions of the circular sequence) were merged to generate a circular consensus sequence, and the resultant sequence adjusted (by shifting sequence from the end to the start of the circular genome) so that the first base in the genome was set to the beginning of the COX1 gene (following the convention of OGRe ^27^, see http://drake.physics.mcmaster.ca/ogre). A final round of error correction was carried out on the circularised genome using Bowtie2-aligned reads from ERR063640 (see Supplementary File 4), producing a final mitochondrial genome length of 13,355 bp. The original 19 kbp contig thus contained about 6 kbp of duplicated sequence. MinION reads were mapped to the assembled genome to identify variants not present in the WTSI reads.

### Comparison of WTSI and MIMR *N. brasiliensis* strains

**Figure 1.**
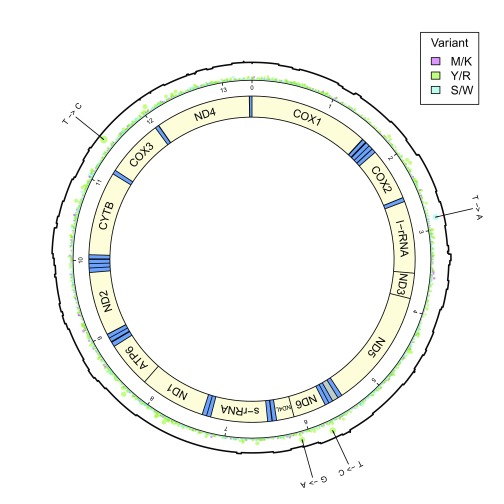
Diagram of mtDNA with mapped MinION read coverage. Gene regions are displayed in this circular mitochondrial DNA diagram in yellow, with tRNA regions in blue. The AT-rich region between the ND5 and ND6 genes is shaded grey. A combined coverage/variant plot is also displayed, showing MinION read coverage (in black), and base-called transition, transversion, and complementary variants (in chartreuse, magenta and cyan, respectively). Variant differences between Wellcome Trust Sanger Institute and Malaghan Institute of Medical Research strains of *Nippostrongylus brasiliensis* are indicated on the perimeter of the diagram.

After remapping the original R9 MinION reads back to the assembled and corrected genome with GraphMap ^28^, four locations were found with variant calls that contributed to more than 50% of the read coverage. Three of these variants involved transition mutations: *T → C* at 5742, *G → A* at 6102, and *T → C* at 11460. One additional complementary mutation was found: *T → A* at 2860 (see Figure 1).

### Mitochondrial genome annotation

**Figure 2.**
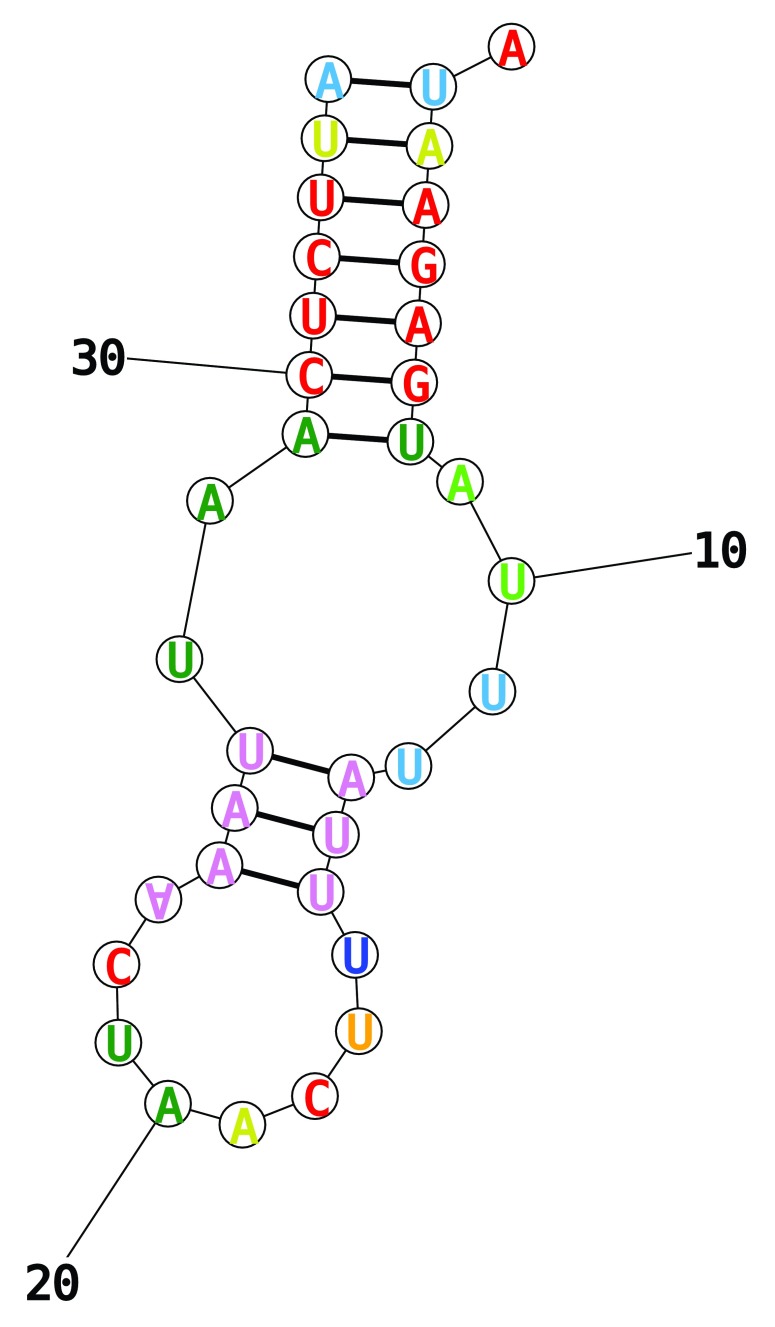
Predicted truncated tRNA structure. RNA structure for the truncated tRNA between ND4 and COX1, predicted by *RNAstructure*.

Approximate gene boundaries were determined by a local NCBI BLASTx search, mapping the contig to mitochondrial protein sequences from *Necator americanus* (see Table 1; Supplementary File 5 and Supplementary File 6). Regions between genes were then scanned using Infernal cmscan ^29^ to identify exact tRNA gene boundaries and codon sequences (see Table 2). The amino acid associated with each tRNA was identified using BWA-MEM to map annotated tRNA sequences from *Oesophagostomum columbianum*, *N. americanus*, *Strongylus vulgaris*, and *A. duodenale*. One tRNA region found by cmscan (between the ND4 and COX1 genes) could not be matched to any existing tRNA sequences. When this sequence was fed into *RNAstructure* ^30^, the predicted secondary structure had no T-loop or D-loop, and an anticodon loop of 8 bases ( Figure 2). The anticodon for this structure pairs with one of the two most common gene start codons (i.e. ATT), and could potentially pair with the other most common start codon through a wobble A-A pairing on the third base (see 31).

**Table 1.** mtDNA gene regions. Predicted gene features from the *Nippostrongylus brasiliensis* mitochondrial genome. Stop codons that end in hyphens (-) are completed by the addition of polyA sequence.

Start	End	Name	Start Codon	Stop Codon
1	1575	COX1	ATT	TAG
1820	2514	COX2	TTG	TA-
2571	3522	l-rRNA	—	—
3523	3857	ND3	ATA	TAA
3858	5438	ND5	ATT	TTA
5498	5578	AT-rich	—	—
5689	6123	ND6	ATA	TAA
6124	6356	ND4L	ATT	TA-
6474	7223	s-rRNA	—	—
7339	8206	ND1	ATT	T--
8207	8806	ATP6	ATT	TAA
9003	9842	ND2	ATT	TAA
10076	11186	CYTB	ATA	T--
11242	12007	COX3	ATA	T--
12062	13291	ND4	GTT	TAA

**Table 2.** mtDNA tRNA sites. Predicted tRNA sites in the *Nippostrongylus brasiliensis* mitochondrial genome. One truncated tRNA site between the ND4 and COX1 genes (detected by *cmscan*) could not be fully annotated.

Start	End	Amino Acid	Codon
1589	1638	Cys	GCA
1649	1705	Met	CAU
1706	1760	Asp	GUC
1764	1819	Gly	UCC
2517	2570	His	GUG
5439	5497	Ala	UGC
5579	5633	Pro	UGG
5634	5688	Val	UAC
6356	6411	Trp	UCA
6417	6473	Glu	UUC
7224	7278	Asn	GUU
7279	7338	Tyr	GUA
8818	8880	Lys	UUU
8890	8944	Leu	UAA
8944	8997	Ser	UCU
9843	9901	Ile	GAU
9902	9959	Arg	ACG
9959	10013	Gln	UUG
10022	10075	Phe	GAA
11187	11241	Leu	UAG
12008	12057	Thr	UGU
13322	13355	—	*AUU*

Precise gene start boundaries were determined by mapping open reading frames (ORFs) between the tRNA genes (codon translation table 5: Invertebrate Mitochondrial) with NCBI SmartBLAST ( https://blast.ncbi.nlm.nih.gov/smartblast/smartBlast.cgi?CMD=Web). Stop boundaries were determined by looking for plausible in-frame stop sequences surrounding the end region of matching SmartBLAST hits. The boundaries for the ribosomal RNA genes were determined by a BLAST search against the four previously compared parasite species. Finally, the AT-rich region was identified as the region between tRNA-Ala and tRNA-Pro.

### Phylogenetic analyses

**Figure 3.**
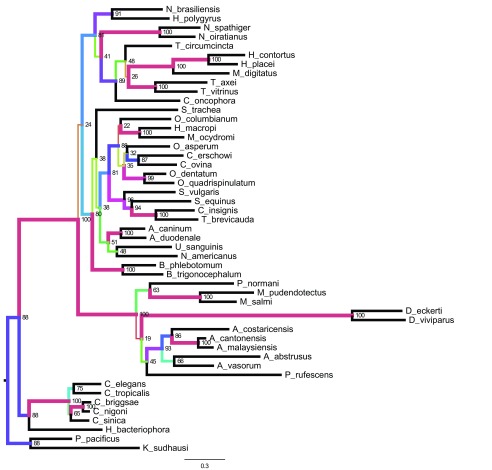
Phylogenetic tree for mtDNA. Phylogenetic tree based on evidence from three mitochondrial-encoded genes: *cytochrome oxidase 1*, *l-rRNA*, and *cytochrome B*. This tree demonstrates sequence similarities for 47 species from the *Rhabditida* together with two outgroups ( *Pristionchus pacificus* and *Koerneria sudhausi*). Branch lengths are nucleotide substitutions per bp. Nodes are labelled with sub-sequence deletion bootstrap values. Branch colours and width are representative of bootstrap proportion.

We identified orthologues of cytochrome oxidase 1 (COX1), cytochrome B (CytB), and the large ribosomal RNA subunit (l-rRNA) in other rhabditid nematodes using BLAST, and collated a dataset from 49 taxa. Nucleotide sequences were aligned using clustalo ^32^, trimmed with trimAL, and phylogenies estimated using RAxML using the GTRGAMMA model. Bootstrap values were calculated from 100 iterations. Figures were generated using FigTree v1.4.2 ( http://tree.bio.ed.ac.uk/software/figtree/). The *Nippostrongylus brasiliensis* sequences were placed within Strongylomorpha, as expected, and *N. brasiliensis* was found to be sister to *Heligmososmoides polygyrus*, a finding in keeping with morphological systematics. Many internal nodes have very low bootstrap values, suggesting either low or conflicting signal in the data. Some groups were well supported, but these tend to be within rather than between genera. Overall the tree conforms to the classical morphological and global molecular phylogenies of the suborder, but cannot stand as indicators of those relationships independently ( Figure 3).

Park and colleagues ^32^ used whole mitochondrial genomes (i.e. all 12 protein coding loci) to develop a phylogeny of Nematoda, with the goal of analysing the placement of some unusual mitochondria from Ascaridia species, but including many strongyles. Our analyses are largely congruent with theirs, albeit with lower support (as noted above).

### Read mapping

The template and complement raw signal from the MinION reads mapped by GraphMap ^28^ were extracted from the FAST5 files, and sorted into four groups:

1. Template sequence, mapped to coding strand
2. Template sequence, mapped to non-coding strand
3. Complement sequence, mapped to coding
4. Complement sequence, mapped to non-coding

A summary of mapping counts can be found in Table 3. Reads where the template fragment mapped to the non-coding strand were about two-thirds that of coding strand-mapped reads, with a similar proportion of reads distributed between the template and complement read fragments.

**Table 3.** mtDNA read groups. Statistics for the four different read mapping groups, showing reads that mapped to the *Nippostrongylus brasisiliensis* mitochondrial genome with over 50% coverage.

Direction	Strand	Count	Mean Length
Template	Coding	26	5.0 kbp
Complement	Non-coding	25	4.8 kbp
Template	Non-coding	17	5.3 kbp
Complement	Coding	16	5.1 kbp

### Event mapping

**Figure 4.**
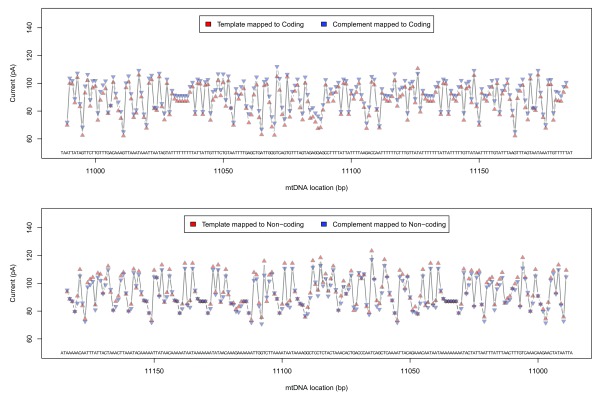
Ideal event plot, CytB gene tail. Ideal event trace for 200 pentamers at the tail end of the Cytochrome B gene. The complement sequence has a slightly lower current than the template sequence for reads mapped to the coding strand, and also a slightly lower current for reads mapped to the non-coding strand.

Event information (generated by the ONT cloud base caller Metrichor dragonet, version 1.22.4) was extracted for these sorted reads, and per-group median event currents were calculated for each pentamer found in the reference mitochondrial genome. An ideal signal trace of the mitochondrial genome was generated using these statistics for the four different signal groups (see Figure 4; Supplementary File 7).

Median complement events mapped to coding strand pentamers had a slightly higher event current when compared to template events (median difference = 3.94 *pA*, 90% range: 1.2 *∼* 6.7, *M AD* = 1.53), and were lower in events mapped to non-coding pentamers (median difference = −2.08 *pA*, 90% range: −5.7 ∼ 1.6, *M AD* = 2.93).

The median signal level for pentamers found in the *N. brasiliensis* mitochondrial genome has a very strong positive correlation between read direction for the coding strand ( *r* = 0.982, 90% range: 0.980 ∼ 0.984) and the non-coding strand ( *r* = 0.974, 90% range: 0.972 ∼ 0.978), whereas there is weaker negative correlation between strands for the template direction ( *r* = −0.67, 90% range: −0.70 ∼ −0.63) and the complement direction ( *r* = −0.66, 90% range: −0.69 ∼ −0.62).

### Raw signal mapping

**Figure 5.**
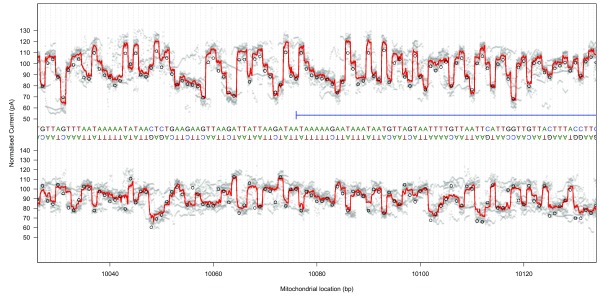
Raw signal plot, CytB gene head. Raw signal plot for 100 bases at the start of the Cytochrome B gene for template read directions (top) and complement read directions (bottom). Median raw signal current is shown as a thick red line, with individual raw signal observations shown in grey. Ideal event current for the observed pentamers is shown as black circles.

Raw signal traces from both template and complement strands were converted to pA using scaling metadata in the FAST5 files, mapped to the GraphMap-aligned reference base positions using event metadata, and linearly interpolated to 11 samples per base using the R *approx* function (R version 3.3.1). Median signal traces (at a sub-base resolution) were generated by summarising the mapped signal at each interpolated location ( Figure 5; Supplementary File 8).

The event data signal for template sequence mapped to the coding strand was loosely correlated with median raw signals in the middle of the interpolated region ( *r* = 0.52, 90% range: 0.51 ∼ 0.53), with other read groups demonstrating lower correlations ( *r* = 0.29 ∼ 0.44). This correlation disappeared when shifting the compared signal by one base in either direction ( *r* = 0.03 ∼ 0.09).

## Discussion

Using a long-read assembler, and three passes of error correction with publicly-available data, we have created a full-length, error-free, *de novo* assembly of the mitochondrial genome of *N. brasiliensis*. This genome has been annotated with gene and tRNA boundaries, and compared with other related parasite species. An additional preliminary “electrical” annotation was generated from mapped nanopore read sequences.

### Mitochondrial genome assembly

Low-cost long-read sequencing has made possible full-length assemblies of a number of different megabase-length genomes from nanopore data alone (e.g 11, 33– 35), so it is not surprising that a full-length mitochondrial assembly was also possible using nanopore reads. The vast wealth of publicly-available data allows fast and low cost assembly, correction, and annotation of genomes, producing high-quality reference sequences that are of great benefit to medical research.

We were able to assemble the *N. brasiliensis* mitochondrial genome from a whole-genome sequencing nanopore dataset, by identifying assembly contigs with high relative coverage. The assembly is of high quality, based on read coverage, mapping of Illumina short reads, and annotation. The gene order is identical to that of *Caenorhabditis elegans* and other strongylomorph nematodes (see 36). Despite this shared structure, there is sufficient variation in sequences between species to generate resolved phylogenies ^32^.

### WTSI assembly of mtDNA

During the final preparation of this paper for publication, the WTSI deposited an annotated mitochondrial genome for *N. brasiliensis* (accession id: AP017690.1). This complements the introduction of the WormBase ParaSite resource for helminth genomics ^9^. While the associated reference for the WTSI *N. brasiliensis* mitochondrial genome is not yet published, it is expected that this mitochondrial genome was assembled using a similar method to the WTSI’s previous work ^37^ (i.e. a reference-based iterative mapping procedure using MITObim ^38^).

The sequence of this assembly differs only in an additional T insertion into a 10 base poly-T tract in the l-rRNA gene. While such polynucleotide tracts are problematic for MinION, the polyT region appears to be polymorphic, with some support for both variants in the WTSI reads (ERR063640). In addition the WTSI annotation excludes the AT-rich region.

### MinION whole genome sequencing data from a metazoan can be used for taxon identification

At the time of sequencing, no mitochondrial genome for *N. brasiliensis* was available. We thus explored the utility of the MinION data in species identification. As the mitochondrial genome is at a higher molarity than the nuclear genome, low-coverage sequencing of a target genome can yield deep coverage of the mitochondrion. Assembly of this replicon, and then analysis in a phylogenetic context was successful in placing *N. brasiliensis* in the Strongylomorpha. We suggest that this approach would be a useful technology for identification of unknown specimens in clinical practice, biosurveillance or biodiversity research programmes. In addition the nanopore electronic signal of the mitochondrial sequence could be used in a “read until” approach ^39^ to diagnosis, using live monitoring to identify reads that likely derive from this, or a very similar genome. Usually, identification through sequencing is applied to amplification of specific target loci in a specimen or sample, an approach known as DNA barcoding. Direct sequencing of the whole genome of a specimen on MinION would allow both barcoding and produce additional sequences that could be used for, for example, population genetic diversity analysis.

### Nanopore read analyses

Nanopore reads were separated into four different read groups to provide information that could be used to establish whether or not there are different sequencing features associated with template and complement strands. In general, the coding and non-coding strands had similar electrical profiles, as demonstrated by the event data (e.g. see Figure 4).

As this investigation is the first attempt to categorise the electrical properties of a complete mitochondrial genome, errors in the data analysis (e.g. due to incorrect mapping, low read coverage, and incorrect scaling parameters) cannot be excluded as an explanation for the difference in current that were observed between event data and raw signal. A comparison of raw signal current to the ideal current suggests that the pentamer model is probably sufficient to fully describe variation in signal in the mitochondrial genome. Although correlation between the signal and the ideal pentamer model is low for all four sequencing groups (template coding, template non-coding, complement coding, complement non-coding), this variation could be explained by errors in the raw signal mapping process, and other alternative mapping techniques (e.g. nanoraw ^40^) may give better performance for linking raw signal to sequenced bases.

It is possible that the observed difference between the raw and ideal event signal may be due to methylation and other epigenetic modification of the mitochondrial genome. Methylation is a known feature of mitochondrial DNA (see 41), and methylation patterns can be observed as changes in the nanopore electrical signal ^42^. Due to the lack of information about epigenetic patterns from *de novo* nanopore sequencing, this dataset is provided without additional epigenetic analysis as a source of discovery for other researchers.

## Conclusions

The data presented here have been created from a minimally-prepared whole-genome DNA from *N. brasiliensis*, combining nanopore reads with publicly-available datasets. Using non-targeted sequencing, we have been able to generate a fully-annotated (gap-free) mitochondrial genome, with an initial electrical signal annotation having a resolution that is finer than a single base. The analysis proves that the efficiently MinION-generated mitochondrial genome of *N. brasiliensis* is of high enough quality for phylogenetic use.

We hope that the procedures discussed here will be sufficient to guide other researchers in annotating mitochondrial genomes and generating consensus signal traces, and that these data will contribute more generally towards improving the sequence base calling algorithms in the future for devices that implement sequencing by observation.

## Data availability

The data referenced by this article are under copyright with the following copyright statement: Copyright: © 2017 Chandler J et al.

Sequences have been deposited into NCBI Genbank, with accession number KY347017. Reads used to produce this assembly are associated with BioProject PRJNA328296. The assembly was error corrected using Illumina reads from a Wellcome Trust Sanger Institute sequencing run ( ERR063640).

The *mpileup2proportion.pl* custom script that was used for error-correcting nanopore reads using Bowtie2-mapped short reads, as well as for generating count data for the read coverage plot, is available from David Eccles’ github repository (DOI, 10.5281/zenodo.164193) ^43^. Read mapping group statistics were generated using the *fastx-grep.pl* and *fastx-length.pl* scripts also from this repository. These scripts have also been included here as a supplementary file ( Supplementary File 9).

## Supplementary files

**Supplementary File 1** Original single-contig assembly generated by Canu v1.3.

.

**Supplementary File 2** ERR063640 reads mapped to Canu-assembled genome, digitally normalised to one read per base.

.

**Supplementary File 3** ERR063640 reads mapped to the first error corrected-genome, digitally normalised to one read per base.

.

**Supplementary File 4** ERR063640 reads mapped to the second error corrected-genome, digitally normalised to one read per base.

.

**Supplementary File 5** BED-format file of discovered mtDNA features (prior to correction of boundaries following protein translation).

.

**Supplementary File 6** FASTA file containing subsequences of the mitochondrial genome representing discovered features.

.

**Supplementary File 7** Event-level data aggregated for each base in the mitochondrial genome, including ideal current derived from pentamer signals.

.

**Supplementary File 8** Data file containing interpolated raw signal-level data.

.

**Supplementary File 9** Compressed file containing all Perl and R scripts used for data processing and analysis.
