# Supplementary material for: Annotated mitochondrial genome with Nanopore R9 signal for Nippostrongylus brasiliensis
Source: F1000Res. 2017 Jan 19;6:56. [Version 1] doi: 10.12688/f1000research.10545.1 (PMC5399971; doi:10.12688/f1000research.10545.1)
Supplement: Peer review history

# Peer review: Referee response for version 1

**DOI:** 10.5256/f1000research.11363.r21379

**Published:** 2017-04-12

**Authors:** Jianbin Wang

**Affiliations:** 1 Department of Biochemistry and Molecular Genetics, University of Colorado School of Medicine, Aurora, CO, USA

In this manuscript, Chandler *et al *described in detail how they used the Nanopore sequencing technology to assemble the mitochondrial genome of Nippostrongylus brasiliensis. They also annotated the mitochondrial genome and did phylogenetic analysis among a selected group of nematodes. In addition, they characterized the Nanopore sequencing features for this genome. Overall, the authors have demonstrated that they can produce the complete mitochondrial genome from their Nanopore sequencing dataset. 

 The authors were able to recover the mitochondrial genome from a genomic DNA library due to the much higher copy number (often hundreds or thousands of times) of the mitochondrial DNA when compared to the nuclear genome. This approach has also been extensively used to recover mitochondrial and chloroplast genomes in whole genome shotgun libraries. In principle, it should work for any type of sequencing technology. The Nanopore sequencing technology is relatively new and is still fast evolving. In this case the technology does not seem to me to have a clear advantage over the Illumina or other sequencing approaches on mitochondrial genome assembly. In additional, the authors eventually used the Illumina data to do the error correction to make the final assembly. Nevertheless, the authors have presented a complete genome assembled from a combination of Nanopore and Illumina data with a full description of how they did this.

 Not considering the novelty or significance of the work, I think the mitochondrial genome is properly assembled and annotated. The results are clear and the manuscript is well written.

I have read this submission. I believe that I have an appropriate level of expertise to confirm that it is of an acceptable scientific standard.

# Response

**Published:** 2017-04-12

**Authors:** David Eccles

**Affiliations:** Malaghan Institute of Medical Research, New Zealand

Thank you very much for your review of our paper on mitochondrial genome sequencing with the MinION sequencer. We are currently working on updating the paper as per the reports of Christian Rödelsperger and Matthias Bernt, and intend to deliver a full response to them at that time.

 This paper was intended as a stepping stone for investigating techniques that could be used to assemble a parasite genome from *unamplified* genomic DNA using the MinION. We discovered that the run yield in this case was not sufficient for assembling the entire *N. brasiliensis* genome, but being able to assemble a *mitochondrial* genome as a single contig has given us confidence that the technology is capable of improving on the existing Illumina-derived whole genome assembly. We did not intend to wow the world with this paper, rather it was an attempt to demonstrate methods and show how easy and quick it can now be to assemble a genome. Thank you for understanding this aspect of our paper.

 At the time of sequencing, the base-calling software was not sufficiently accurate to generate a reliable sequence at a single base level. Understanding this, we used MinION reads for scaffolding, and Illumina reads (from a different strain) to correct the abundant base call errors. This approach has allowed a relatively cheap and fast assembly of the mitochondrial genome, such that comprehensive phylogenetic analyses can be carried out on the mitochondrial genes.

 As you have mentioned, the nanopore sequencing technology is evolving fast. It is likely the case that updated base-calling software has improved base calling accuracy sufficiently that this approach can be carried out using MinION reads alone. I would like to carry out additional investigations on these data to discover if that is indeed the case, but would rather hold off on that until after we have published our attempts at whole genome assembly. Regardless, the mitochondrial sequences (including raw signal) are available for anyone else to determine themselves whether or not a high-quality MinION-only assembly is possible using re-called (but otherwise identical) nanopore sequence data.

# Peer review: Referee response for version 1

**DOI:** 10.5256/f1000research.11363.r21560

**Published:** 2017-04-11

**Authors:** Christian Rödelsperger

**Affiliations:** 1 Department for Evolutionary Biology, Max-Planck-Institute for Developmental Biology, Tübingen, Germany

The manuscript by Chandler *et al *describes the sequencing, assembly and annotation of the Nippostrongylus brasiliensis mitochondrial genome using Nanopore sequencing technology. The comparison of the resulting assembly with other N. brasiliensis data from the parasite sequencing initatitive of the Sanger Institute and also with mitochondrial genomes from other nematodes support that the produced assembly is of high quality.

 In general, the structure of the article is a bit unusual. Methods and Results sections are combined, each part has multiple subsections that are not really connected. Some parts of the paper deal with the mitochondrial genome of N. brasiliensis other parts focus on very specific aspects of Nanopore sequencing. I would recommend to concentrate on the  mitochondrial genome of N. brasiliensis and keep the nanopore-specific questions for a separate methodological paper.

**Section: Introduction**

 The Introduction basically describes the lifecycle of N. brasiliensis and the mode of infection. The authors might consider writing a more general introduction about nematodes, parasites, .. that it is important to study these parasites to develop treatments. In addtion, there are multiple related parasites that are later part of the phylogenetic analysis. It would be good to give some information about those ones as well. e.g. what are their hosts?

**Section: Current reference genome**

 Please provide the Genbank entry for the NCBI reference genome or provide the assembly that has been used for this study as supplemental data. Otherwise, it will be hard to reproduce the results.

**Section: Scientific justification**

 please explain what a "read until” methodology is and provide some reference for the use of ONT MinION in studies of infectious disease outbreak.

 Is the N. brasiliensis isolate that was used for sequencing have a strain ID? If yes, please specify and at least register a biosample for it and give the accession number. Was it the same isolate that was used for the NCBI reference genome.

**Section: Whole-genome assembly with Canu**

 How much sequencing data was obtained? Please provide some more details about the assembly results. How many Contigs, total size.

 For readers, that would like to use Nanopore technology to sequence their genomes it would be interesting to compare the quality of of the mitochondrial genome with nuclear contigs.  I guess, that the lower coverage of nuclear contigs should also result in higher number mismatches with regard to the reference genome. A major finding of the paper could be that based on current nanopore technology, it only makes sense to do the multicopy mitochondrial genome. Such a statement could help people to plan their projects.  

**Section: Error correction and circularisation**

 How many sites had to be corrected. Error correction only makes sense, if the WTSI data is from the same isolate. Please clarify if this is the case. If it is the same isolate, there does the 2% mismatches come from in the "Whole-genome assembly with Canu" section.

**Section: Mitochondrial genome annotation**

 "The amino acid associated with each tRNA was identified using BWA-MEM to map annotated tRNA sequences from Oesophagostomum columbianum, N. americanus, Strongylus vulgaris, and A. duodenale." Using BWA-mem to annotate tRNAs from other species sounds unusual. Do you have a reference where the performance of this methodology has ever been evaluated?

**Section: Phylogenetic analyses**

 Please provide more information about the alignment, how many sites? amount of missing data?   Please provide references for what is called "the classical morphological and global molecular phylogenies"

**Section: Read mapping/  Event mapping /Raw signal mapping  (Table 3, Fig 4 and 5)**

 These sections seem to examine very specific aspects of the Nanopore sequencing technology and do not add any addtional insights for the presented mitochondrial genome. I also have problems in understanding what kind of questions are asked. It seems to me, as if the authors try to examine whether Nanopore data has a preference for the template or complement strand or whether there is a bias for coding or non-coding sequences. How well the sequencing signal corresponds to the basecalls in the final assembly and what features correlate with variation in sequencing signals. The presented results are not conclusive (no statistical tests have been done to assess the significance of the results)  and are not really related to the rest of the manuscript. I would recommend to use this and other comparable data for a separate more methodological paper. One additional feature that could be tested would be how differences raw and ideal event current, sequencing coverage  depend on GC content.

**Minor comments**

**Section: DNA extraction and library preparation**

 The first paragraph should probably labeled as "Worm culturing" or something else. It has nothing to do with DNA extraction or library preparation

**Section: MinION sequencing**

 "sequenced at 60 bases per second with a yield of about 200 Mb" does that mean per sequencing run?

 This section sounds a bit like a promotion of MinION sequencing. I would recommend to reduce it only to the parts that are relevant for the current paper.

 high through-put sequencing -> high-throughput

 I wonder why the title has to have the information that R9 signal has been used. Probably most readers have heard about Nanopore sequencing but do not have a clue what R9 signal is. I would recommend to put this detailed information into the methods section but remove it from the title.

I have read this submission. I believe that I have an appropriate level of expertise to confirm that it is of an acceptable scientific standard, however I have significant reservations, as outlined above.

# Response

**Published:** 2017-04-12

**Authors:** David Eccles

**Affiliations:** Malaghan Institute of Medical Research, New Zealand

Thank you very much for your review of our paper on mitochondrial genome sequencing with the MinION sequencer. We are currently working on updating the paper as per your report (and the report of Matthias Bernt), and will deliver a full response once the next revision of the paper is ready.

# Peer review: Referee response for version 1

**DOI:** 10.5256/f1000research.11363.r19519

**Published:** 2017-02-20

**Authors:** Matthias Bernt

**Affiliations:** 1 Department of Computer Science, Leipzig University, Leipzig, Germany

The paper describes the sequencing of the mitochondrial genome of the *Nippostrangylus brasiliensis* with the novel Nanopore sequencing technique. To the best of my knowledge this seems to be one of the first mitogenomes that have been sequenced with this technology. The annotation of the genome and its use

 for phylogeny and taxonomic identification have been discussed.

 Another group has sequenced the genome (including the mitogenome) has been sequenced using another NGS strategy. While this seem unfortunate its actually good for this study otherwise no reference data would have been available for comparison and error correction. I'm missing an analysis of the error rates of the sequencing without the correction that has used the read data from the other study. I'm wondering if the combination of data from MiniON sequencing and short read sequencing strategies might be a good general strategy?

 The paper is well written and needs only a few corrections and additions. Details are given below.

**Abstract:**

 =========

 The term "electrical consensus sequence" might be puzzling for uninformed readers.

**Introduction:**

 =============

 "L3" is also difficult to understand for non experts. Maybe add 'stage'?

 "highquality" missing space

**MiniON sequencing**

 =================

 "R7.3" Can you explain what this means?

 "89% pores" is unclear to me.

 What are "2D reads"?

**Scientific justification**

 ========================

 "strict maternal inheritance": nothing in biology is strict. Check for paternal leakage or doubly-uniparental inheritance.

 The term "read until" methodology is unclear.

**DNA extraction and library preparation**

 ======================================

 Explain the abbreviation PBS

**Error correction and circularisation**

 ====================================

 It needs to be explained what the custom script is doing.

 "Repeated sections of the linear contig were merged... " What happens with true repeats?

 Since not all readers might know the color chartreuse I would suggest to order the colors as in the legend.

**Mitochondrial genome annotation**

 ===============================

 I'm wondering why automatic methods for genome annotation have been ignored. Not saying that the applied approach is wrong.

 When you use cmscan you need to state the used model as well.

 "tRNA... codon sequences" Do you mean anticodon?

 How about non-canonical start codons? How do you define "plausible" in frame stop?

 For the truncated tRNAs there are examples known for Enoplea: see http://dx.doi.org/10.4161/rna.21630 and http://dx.doi.org/10.1016/j.biochi.2013.07.034

**Phylogenetic Analyses**

 =====================

 References for RAxML and trimAL are missing.

**Event Mapping**

 =============

 "Event information" Specify what an event is.

 "per-group" and later on "signal groups" You should reformulate this. Currently its a bit confusing.

 Why pentamer?

 What is an ideal signal trace?

**Raw Signal Mapping**

 ==================

 Has Graph Map been referenced?

**Discussion**

 ==========

 Are you really sure that the sequence is "error free"? In the end of the paper you write that its of "high enough quality...".

I have read this submission. I believe that I have an appropriate level of expertise to confirm that it is of an acceptable scientific standard, however I have significant reservations, as outlined above.

# Response

**Published:** 2017-04-12

**Authors:** David Eccles

**Affiliations:** Malaghan Institute of Medical Research, New Zealand

Thank you very much for your review of our paper on mitochondrial genome sequencing with the MinION sequencer. We are currently working on updating the paper as per your report (and the report of Christian Rödelsperger), and will deliver a full response once the next revision of the paper is ready.
